# Karyological Analysis and DNA Barcoding of Pompia Citron: A First Step toward the Identification of Its Relatives

**DOI:** 10.3390/plants8040083

**Published:** 2019-03-31

**Authors:** Grazia Viglietti, Giulio Galla, Andrea Porceddu, Gianni Barcaccia, Franck Curk, Francois Luro, Grazia Maria Scarpa

**Affiliations:** 1 Dipartimento di AGRARIA Research Unit SACEG, University of Sassari, 07100 Sassari, Italy; graziaviglietti@gmail.com (G.V.); aporceddu@uniss.it (A.P.); 2 Laboratory of Genomics, Department of Agronomy Food Natural Resources Animals and Environment, University of Padova, 35020 Legnaro, Padova, Italy; giulio.galla@unipd.it (G.G.); gianni.barcaccia@unipd.it (G.B.); 3 Unite Mixte de Recherche Amelioration Genetique et Adaptation des Plantes (UMR Agap), Institut National de la Recherche Agronomique (INRA), F-20230 San Giuliano, France; franck.curk@inra.fr (F.C.); francois.luro@inra.fr (F.L.)

**Keywords:** Karyotype, DNA content, ITS sequence, psbA-trnH sequence, trnL intron sequence

## Abstract

Pompia is a citrus fruit endemic of Sardinia, Italy, with an essential oil profile showing outstanding anti-inflammatory and anti-microbic properties. Despite its remarkable pharmaceutical potential, little taxonomic and genetic information is available for this species. We applied flow cytometry and classical cytogenetic techniques to assess the DNA content and to reconstruct the karyotype of several Pompia accessions. Molecular data from plastid DNA barcoding and nuclear DNA sequencing were used to study the genetic distance between Pompia and other citrus species. Flow cytometric estimates of DNA content and somatic chromosome counts suggest that Pompia is a regular diploid Citrus species. DNA polymorphisms of nuclear and chloroplast markers allowed us to investigate the genetic relationships between Pompia accessions and other Citrus species. Based on DNA polymorphism data we propose that Pompia is a very recent interspecific hybrid generated by a cross between *C. aurantium* (as seed bearer) and *C. medica* (as pollen donor). Our findings pave the way for further and more specific investigations of local Pompia germplasm resources that may help the preservation and valorisation of this valuable citrus fruit tree.

## 1. Introduction

Citrus is a genus of flowering trees and shrubs belonging to the family Rutaceae. Species belonging to this genus have very small mitotic chromosomes (1.0–4.0 μm) and most of them are similar in their morphology [1]. The chromosome number was established by Frost [1] as 2n = 2x = 18; diploidy is widespread in the genus, with the exception of some cultivated polyploids [2,3] such as 2n = 3x = 27 for *C. aurantifolia* and *C. latifolia* [4,5] and 2n = 3x = 27 or 4x = 36 in *C. limonia* [1].

Citrus species comprehend many interspecific hybrids and several economically important crops such as oranges, lemons, pomelo, limes and grapefruit [6]. While many Citrus hybrids have gained wide diffusion around the world, some others are still almost unknown to most consumers and their cultivation is restricted to small orchards as unique endemism [7]. Since the initial definition of the genus by Linneaus in 1753, the taxonomic classification of Citrus species has proved particularly controversial.

Several features of Citrus biology and cultivation methods hampered the univocal definition of several species [8]. These features include a high level of sexual interspecific compatibility within the genus and clonal propagation occurring either by grafting or apomixis [9]. Polyploids, which are frequently found within the genus, may be originated from events of chromosome complement duplication occurring in a somatic cell of the nucellus before the onset of adventitious embryony (e.g., sporophytic apomixis), in the case of tetraploidization [1] or from an unreduced egg cell giving rise to unbalanced BIII hybrid upon fertilization, which is the main way of triploid formation [2]. Some other factors, such as interspecific hybridization, ploidy level, atmospheric temperature during flowering period and the mono- or poly-embryonic nature of Citrus may also influence to the frequency of polyploid progenies [10,11].

The complexity of Citrus taxonomy can be somehow exemplified by considering, for instance, the great variability in the number of species recognized by the two major systems adopted so far: Swingle and Reece consider 16 species [12] while Tanaka identifies 156 species [13]. However, despite the difficulties in reaching a consensus in the taxonomy at the species level, most authors agree on the origin of most cultivated forms.

Webber [14] and Calabrese [15] proposed that Citrus spp. originated in the tropical and subtropical regions of Southeast Asia and then spread to other continents. Scora [16] and Barret Rhodes [17] suggested that there are only three basic species between cultivated citrus: *Citrus medica* (citron), *Citrus reticulata* (mandarin) and *Citrus maxima* (pummelo). According to these authors, the other genotypes derived from multiple hybridization events that occurred between these three species during the long history of cultivation and dispersion among many countries worldwide [7]. Remarkably, the ancestral origin of three basic Citrus species: citron, mandarin and pummelo, along with their relative contribution to the breeding of lemons, limes, orange and grapefruit has been recently confirmed by sequencing data [8].

*Citrus* sp. ‘monstruosa’ (NCBI:txid1430428), also known as Pompia, is a citrus fruit endemic of Sardinia, Italy [18]. From a morphological point of view, its fruit is characterized by a rough skin with a disagreeable aspect, which possibly determined its initial taxonomic classification (i.e., *Citrus monstruosa* [18]). Pompia’s essential oil profile is rich of oils which are credited of a strong anti-inflammatory and antiseptic activity [19,20,21]. While the fruit pulp is not edible, its skin has been used for centuries to prepare Sardinian traditional cakes and liquors [22]. Although the cultivation and diffusion of Pompia-derived products is currently restricted to niche productive and socio-cultural areas in Sardinia [22], the development of efficient tools useful to assess the genetic authenticity of Pompia-derived products is becoming a relevant issue.

Chloroplast DNA barcoding is a molecular system useful to identify plant species and, to a lesser extent, to verify the distinctiveness of genotypes and relatedness among genotypes within and between populations [23]. It was initially developed to address taxonomic uncertainties arising from the restriction of morphological features to a particular life stage [24], characters homoplasy [25], missing body parts [26] or poor sample preservation. Compared to techniques for nuclear DNA genotyping, such as restriction fragment length polymorphism (RFLP), amplified fragment length polymorphism (AFLP), simple sequence repeat (SSR) and other PCR-derived markers, it allows to identify the plant genus and/or species by obtaining a short DNA sequence from known target regions of the chloroplast genome (cpDNA) and comparing it with databases of orthologous sequences from species of established identity. Since the adoption of DNA barcoding [27,28,29,30], cpDNA sequence polymorphism has been investigated in several Citrus species [31]. Although the conclusions outlined by these studies proved often conflicting, their findings confirmed the efficacy of few cpDNA markers (i.e., barcodes) in species identification and/or validation.

This research is aimed at investigating the involvement of some Citrus species in the Pompia’s genetic origin by using a DNA barcoding-based strategy, along with karyological and flow cytometrical analyses. In particular, the cpDNA barcodes corresponding to the intergenic region psbA-trnH and the trnL genic intron along with the internal transcribed spacer (ITS) nuclear locus were investigated by Sanger sequencing in order to clarify the phylogenetic relationships of Pompia with respect to other species belonging to the same genus.

## 2. Results and Discussion

### 2.1. Pompia Is a Regular Diploid Citrus

Karyo-morphometric features of Pompia’s chromosomes were visualized in squashes of colchicine treated root tips of several Pompia accessions.

**Figure 1:**
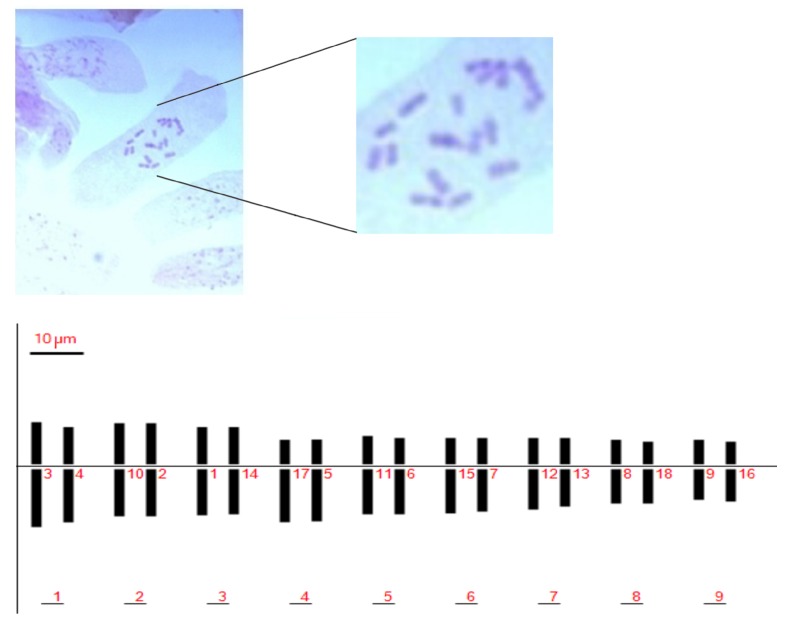
Pompia chromosomes in squashes of colchicine treated root tips (top panel); Pompia deduced karyotype (bottom panel).

No significant differences in measured karyological parameters were visualized among the analyzed accessions. Chromosome types were classified based on their arm ratio according to Levan et al. (1964) [32]. We identified a total chromosome number of 2n = 2x = 18, sixteen of which were classified as metacentric and two as sub-metacentric (Figure 1 and Table 1). The Pompia karyogram, as reconstructed by the Karyotype software [33], is shown in Figure 1.

The ratio between the largest and the smallest chromosomes was found equal to 1.82. The long arm of the most asymmetric chromosome was more than twice longer than the short arm. According to these parameters, we assigned Pompia to the 2A class of karyotype asymmetry class [34].

The Total Haploid Length of chromosome (THL) was estimated as 162.25 μm. The centromeric asymmetry and mean centromeric asymmetry were 9.94 and 18.54, respectively. The chromosome lengths showed a rather uniform distribution and the coefficient of variation of chromosome length (CVCL) was estimated in 18.85. For a complete list of estimated karyotype asymmetric indices see Supplementary File 1.

**Figure 2:**
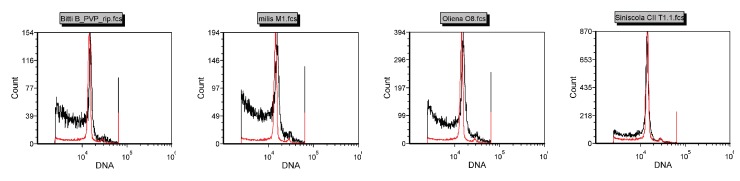
Flow citometry of Pompia accessions. *C limon* was used as internal reference (red line). *X*-axis represent the fluorescent intensity of DAPI-staining and *Y*-axis the counts of measured nuclei.

The nuclear DNA content of Pompia accessions was measured by flow cytometry analysis using *C. limon* (1C = 0.40; pg [35]) as diploid reference. No significant differences between the DNA content estimates of *C. limon* and Pompia accessions were detected (Figure 2).

Altogether, karyological and flow cytometrical data suggest that Pompia is a regular diploid citrus fruit tree.

### 2.2. Evolutionary Divergence of Pompia and Citrus Species Based on Single Nucleotide Polymorphisms at Nuclear or Cytoplasmic Loci

Molecular investigations were carried out on 10 Pompia accessions (Table 2) along with accessions of the three basic Citrus species: *C. medica*, *C. maxima*, *C. reticulata* and five secondary species, including *C. bergamia*, *C. aurantium*, *C. micrantha*, *C. sinensis* and *C. limon* (Table 2), which based on available morphological data [18], could be considered among the most informative for Pompia taxonomic investigations (Table 2). The citron Rhobs el arsa (ICVN0110244) is a *Citrus* spp. accession morphologically similar to Pompia.

Preliminary investigations performed using the cpDNA sequences available in BOLD v4 [37] for the two canonical barcodes rbcL and matK revealed a very low nucleotide variation and polymorphism rate between *C. medica*, *C. limon* and *C. aurantium* [27,28,29,30]. We therefore focused our investigations on the intergenic (psbA-trnH) and intronic (trnL) cpDNA regions, together with the nuclear ITS locus, chosen as an nuclear marker for studying nucleotide diversity. The cpDNA amplicons ranged from 442 bp to 468 bp and from 530 bp to 542 bp, for the psbA-trnH and trnL-intron barcodes, respectively. The size of ITS amplicons ranged from 533 bp to 564 bp. Overall, the mean genetic distance was equal to 0.007 for psbA-trnH and 0.002 for trnL-intron sequences. The mean genetic distance for the ITS marker sequences was 0.010. The alignments of nucleotide sequences for the nuclear ITS and cpDNA regions are provided in the Supplementary Files 2 and 3, respectively. Pairwise estimates of evolutionary divergence between taxa are shown in Table 3.

As expected, the three Citrus basic species scored high levels of evolutionary divergence [38]. *C. medica* (p: 0.004) was the closest to Pompia, followed by *C. maxima* (p: 0.010) and *C. reticulata* (p: 0.022). Since ITS sequences in several Citrus spp. accessions were associated with heterozygous loci, we subtracted the mean distance within groups from the average distance between groups (Table 3). By doing so, the average net distance of Pompia was estimated as 0.019 from *C. reticulata* and 0.007 from *C. maxima*, while the net distance between Pompia and *C. medica* was equal to 0.001. In relation to secondary Citrus species, Pompia showed the lowest level of ITS sequence divergence from the citron Rhobs el arsa (average net distance: −0.003) and *C. bergamia* (average net distance: −0.003). The cp-DNA markers did not resolve univocally the genetic relatedness between Pompia and the basic species. The trnL marker identified *C. medica* as the closest basic species to Pompia and based on the psbA-trnH marker this species scored the highest distance from Pompia. *C. maxima* was the closest basic species to Pompia based on psbA-trnH marker while based on the trnL marker this species together with *C. reticulata* proved the most divergent from Pompia among the basic species.

Regarding the pairwise comparisons involving Pompia and secondary Citrus species, both cpDNA marker loci indicated complete identity between Pompia and Rhobs el arsa, *C. bergamia*, *C. aurantium* and *C. limon* and an average distance of 0.002 with *C. sinensis*. The distance between Pompia and *C. micrantha* was 0.002 for trnL and 0.014 for psbA-trnH (Table 4). The ITS multiple sequence alignment displayed a stretch of 29 contiguous gaps starting from the nucleotide position 213 in sequences amplified from Pompia, *C. limon*, *C. medica* and *C. bergamia* (see Supplemental File 2). These observations prompted us to look for haplotypes discriminating the analysed species. The region corresponding to the ITS1 provided 44 SNVs (Single Nucleotide Variants), 29 of which were In/Dels whereas the region corresponding to the ITS2 revealed 5 SNPs and a single In/Del at position 451 of the alignment (Table 5).

**Figure 3:**
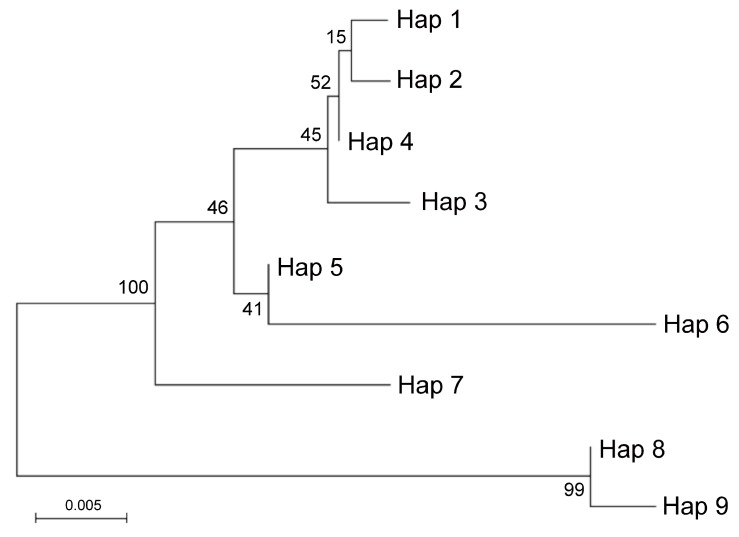
Molecular phylogenetic analysis by Maximum Likelihood method of ITS haplotypes. The percentage of replicate trees in which the associated taxa clustered together in the bootstrap test (500 replicates) is shown next to the branches. Hap1 was identified in Rhobs el arsa, Pompia and *C. aurantium*; Hap2 and Hap3 in *C. sinensis*; Hap4 in *C. maxima*; Hap5 in *C. bergamia*; Hap6 in *C. reticulata*; Hap7 in *C. micrantha*. Hap8 in Pompia, *C. medica*, Rhobs el arsa, *C. bergamia*; Hap9 in *C. limon*.

A total of nine haplotypes were identified, including six species-specific haplotypes and two haplotypes common to multiple accessions. More in detail, Hap1 was identified in all Pompia accessions, Rhobs el arsa and *C. aurantium*, while Hap8 was found in Pompia accessions, Rhobs el arsa, *C. medica* and *C. bergamia* (Table 5). Noteworthy, Hap1 was not identified in basic Citrus species. Furthermore, it is considered that *C. bergamia* (Accession: SRA 212) is heterozygous for the two alleles best described by Hap5 and Hap8, while Rhobs el arsa (Accession: ICVN0110033) share the haplotypes Hap1 and Hap8 with all investigated accessions of Pompia. The occurrence of the observed In/Del in position 213–241 of the sequence alignment, was confirmed in all investigated Citrus spp. accessions, by using specific primers overlapping this In/Del (Table 5). With this respect, it is worth noting that all Pompia accessions were confirmed heterozygous for this locus. Furthermore, consistent amplification profiles were observed for the accessions sharing one or multiple haplotypes with Pompia, namely: *C. medica* (ACC: SRA540, SRA101, Hap8) and Rhobs el arsa (Acc. ICVN0110244; Hap8) , *C. bergamia* (Acc. SRA 612; Hap 5,8) and *C. aurantium* (Acc. ICVN0110033; Hap1) and Rhobs el arsa (Acc. ICVN0110244; Hap1) . The phylogenetic relationships between haplotypes of the ITS locus were summarized in a Maximum likelihood tree (Figure 3).

Two main groups including highly similar haplotypes were identified. One group included Hap8 identified in *C. medica* and Pompia and Hap9 identified in *C. limon*. The other included: Hap1 identified in Pompia, Rhobs el arsa and *C. aurantium*, which clustered in proximity to Hap2 (*C. sinensis*), Hap4 (*C. maxima*) and Hap3 (*C. sinensis*). Hap5 identified *C. bergamia* and Hap6 identified in *C.reticulata* formed a subgroup with lower similarity. Finally, Hap7 specific of *C. micrantha* was in intermediate position between this latter groups. This picture was in substantial agreement with Curk et al. [38]. Based on ITS data it is likely that Pompia inherited the Hap8 from *C. medica* or *C. bergamia* and that Hap1 found in Pompia accessions derived from *C. aurantium* or one of its interspecific hybrids. Moreover, at the level of resolution allowed by these molecular analyses, Pompia and Rhobs el arsa seem to be a case of synonymy.

**Figure 4:**
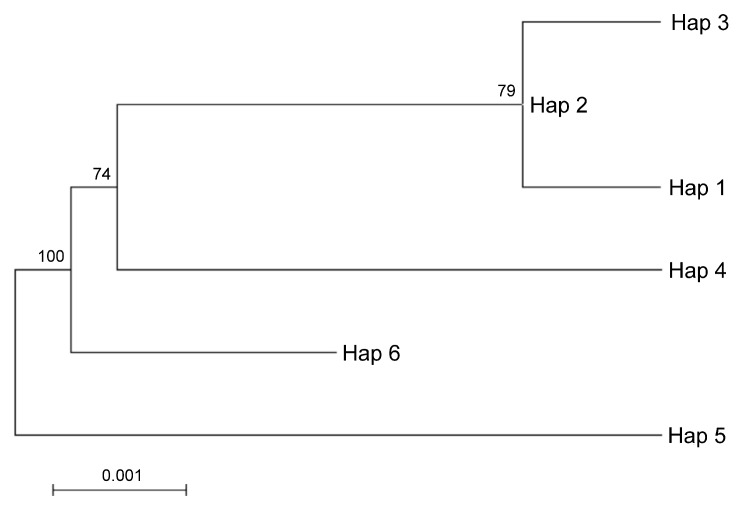
ML tree based on cpDNA haplotypes identified after merging the sequences of psbA-trnH and trnL sequences. Hap1 Pompia and Rhobs el arsa, *C. bergamia*, *C. limon*, *C. aurantium*, Hap2 *C. sinensis*, Hap3 *C. maxima*, Hap4 *C. micrantha*, Hap5 *C. reticulata*, Hap6 *C. medica*. The percentage of replicate trees in which the associated taxa clustered together in the bootstrap test (500 replicates) is shown next to the branches.

Taking into account the maternal inheritance of the chloroplast genome, we integrated the above nuclear DNA findings with chloroplast DNA polymorphisms in order to distinguish between parental species contributions. The analysis of the intergenic region psbA-trnH revealed 51 SNVs, 40 of which were In/Dels. The trnL-intron was less informative as it provided 4 SNPs and 12 In/Dels (for a total of 16 SNVs). Interestingly, the SNVs analysis of the merged chloroplast data set revealed six haplotypes (Appendix A and Supplementary File 3). It is worth noting that a single haplotype, namely Hap1, contained the sequences found in Pompia, together with Rhobs el arsa, *C. aurantium* and the interspecific hybrids originated from this latter secondary species: *C. limon*, *C. bergamia*. Unique haplotypes were found for the remaining species: *C. maxima* (Hap3), *C. sinensis* (Hap2), *C. medica* (Hap6), *C. reticulata* (Hap5) and *C. micrantha* (Hap4). Regarding the relationships among cpDNA haplotypes, the Maximum likelihood tree clustered Hap1 in proximity of Hap3 and Hap2 and far more distantly from Hap 4 (Figure 4). The haplotypes displaying higher evolutionary divergence from Pompia (Hap1) accessions were Hap6 and Hap5, respectively from *C. medica* and *C. reticulata*.

## 3. Conclusions

Pompia citron (NCBItxid: Citrus sp. ‘monstruosa’; also known as ‘sa Pompia’ in Sardinian language) is a Citrus species of unknown origin, endemic of Sardinia island, which possesses an essential oil profile with outstanding anti-inflammatory and anti-microbic properties [17]. To shed some light on the taxonomic origin of this species, detailed cytometric, karyological and molecular investigations were attempted by studying a core collection of Pompia including 10 accessions collected in different geographical areas of Sardinia, together with accessions from the three basic Citrus species and five secondary species which based on morphological traits of fruits are likely related to Pompia. Flow cytometric and karyological investigations demonstrated that Pompia is a regular diploid plant (2n = 2x = 18). Accordingly, the DNA content was estimated in 1C = 0.40 pg, a value in agreement with other estimates reported for the regular diploid *Citrus limon*.

Molecular investigations focused on two cpDNA marker sequences located in non-coding regions (e.g., the intergenic spacer psbA-trnH and the trnL-genic intron), together with the nuclear ITS locus, proved informative in previous studies on Citrus spp. [27,28,29,30]. Among cpDNA markers investigated in this study, psbA-trnH scored the highest number of SNVs, proving to be the most suitable region for discrimination of Citrus accessions, along with the nuclear ITS. These findings are in agreement with that reported by Luo and colleagues [27]. However, psbA-trnH, either alone or in combination with trnL, could not differentiate Pompia from Rhobs el arsa, *C. limon*, *C. bergamia* and *C. aurantium*. Nevertheless, based on our estimates of evolutionary divergence between the investigated accessions, it is considered unlikely that the Pompia originated from either of the two basic Citrus taxa: *C. reticulata* and *C. maxima*. Indeed, nucleotide polymorphisms located in the ITS locus pointed to basic Citrus species *C. medica* and the secondary Citrus species Rhobs el arsa, *C. aurantium* and *C. bergamia* as the closest relatives. Noteworthy, Pompia accessions revealed heterozygosity at the ITS nuclear locus, with two alleles diverging for a 29 bp long In/Del starting from nucleotide position 213 of the alignment. Remarkably, the same heterozygous condition was found in the accessions of Rhobs el alsa and *C. bergamia*. High segmental heterozygosity has already been reported for several hybrid accessions of Citrus [8]. Haplotypes reconstruction for the ITS locus suggests that Pompia inherited one allele from *C. aurantium* or one of its interspecific hybrids, while the second allele most likely derived from *C. medica* or *C. bergamia*.

The maternally inherited cpDNA marker data underlined a high sequence similarity between Pompia chloroplast target regions and those derived from *C. aurantium*, *C. limon*, *C. bergamia* and Rhobs el arsa. Maximum likelihood reconstruction of cpDNA haplotypes showed that Hap3, identified only in *C. maxima*, clustered at little distance from the haplotype that was found in *C. aurantium*, *C. limon*, *C. bergamia*, Rhobs el arsa, and Pompia accessions. Taken together, nuclear and chloroplast DNA polymorphisms and nucleotide variants suggest that all Pompia accessions investigated in this study share the maternal lineage with *C. aurantium* and the paternal lineage with *C. medica*. Based on complete sequence identity for either nuclear and chloroplast DNA markers, we propose that Pompia and Rhobs el arsa, which is very popular in Morocco and in neighbour countries where it takes different names such as Al-zanbu, Koubs el arsa, represent a case of synonymy (i.e., different vernacular names identifying the same genetic and taxonomic entity).

Further studies must be dedicated to ascertaining whether these specimens belong to the same species before reinterpreting the origin and dissemination route of this interesting citrus fruit. The present research represents a first step towards the definition a molecular PCR-based tool useful for efficient genetic traceability of Pompia accessions and its derivative products which could have a potential for nutraceutical and pharmaceutical applications.

## 4. Materials and Methods

### 4.1. Pompia and Citrus spp germplasm

In total, 8 species belonging to the Citrus genus, available in germplasm banks of CRB Citrus INRA-CIRAD, San Giuliano, Corsica (France) were selected as representative of the most likely ancestors of Pompia, based on morphological traits, plant descriptors, and molecular markers [18,19,39]. The two accessions Poncire Commun and Diamante were used to represent the species *C. medica*. One accession identified as Rhobs el arsa (Acc. No ICVN0110244) which appeared to be morphologically similar to citrus was also investigated to better understand hits relationship with Pompia and other citrus species adopted in this study. In addition, 10 accessions of Citrus spp. var. Pompia, were obtained from Sardinia (Bitti, Milis, Oliena and Siniscola). A list of varieties and landraces with information on their origins can be found in Table 2.

### 4.2. Flow Cytometry Screening

Nuclei were isolated from 100 mg leaf tissue by gentile chopping with a razor blade in 0.4 mL of CyStain UV Precise P nuclei extraction buffer (Sysmex Partec GmbH, Gorlitz, Germany) supplemented with 1% *w*/*v* PVP. For each considered sample, 3 replicates were analysed. Following nuclei extraction, the suspension was filtered through nylon tissue of 30 mm mesh width as recommended by the manufacturer. Following the filtration step, 1.6 mL of staining buffer was added to the lysate and the tubes were stored in the dark on ice for 1 h before measurement. The fluorescence intensity of DAPI-stained nuclei was determined using the flow cytometer CyFlow Cube Ploidy Analyser (SysmexPartec GmbH, Gorlitz, Germany) equipped with an UV-Light Emitting Diode (l = 355 nm–375 nm). Data were plotted on a logarithmic scale and calibration of C values was made with nuclei extracted from *C. limon*. Ploidy histograms were quantitatively analysed with the FCS Express 5 Flow software (SysmexPartec GmbH), after manual treatment to exclude noise.

### 4.3. Chromosome Count

Pompia seeds were collected from ripe fruit yielded in November in Siniscola. Seeds were pretreated in 20% sodium hypochlorite for 20 min, and germinated on Petri dishes on tissue paper, incubated at 24 + 1 ℃. Root tips long about 0.5–1 cm were excised, treated with 0.3% colchicine (alkaloid cytostatic) for 4 h at room temperature, then fixed ethanol/acetic acid solution (*v*/*v*, 3:1) overnight at 4 ℃. After 3 washes with distilled water, the root tips were hydrolysed in 1N chlorodric acid for 8 min at 60 ℃, stained in Shiff reagent, and observed under a microscope with a drop of 50% acetic acid. Permanent slides were prepared by dehydration in alcohols progressive series, and then analyzed for karyotype. The evaluation was done with the help of an Axiophot Zeisse microscope, equipped with an Infinity Analyze Lumenera Camera. The output was analyzed trough the Karyotype software [33]. The classification of chromosomes in metacentric (m), sub-metacentric (sm), sub-telocentric (st) and telocentric (t) was made as reported by Levan et al. [32].

### 4.4. DNA Extraction and Amplification

Genomic DNA was isolated from 0.1 g of powdered, frozen, young leaf tissue using the MATAB DNA extraction protocol, described by Cabasson et al. [40]. DNA integrity was estimated by electrophoresis on a 0.8% agarose/TAE gel using the 1 kb Plus DNA ladder (Invitrogen, Carlsbad, CA, USA) as size standards. The purity and quantity of the DNA extracts were assessed with a NanoDrop 3300 spectrophotometer (Thermo Scientific, Bartlesville, OK, USA). Preliminary computational investigations aimed at selecting the optimal chloroplast regions for DNA barcoding were carried out by aligning the available sequences for the chloroplast barcode regions: psbA-trnH, trnL-intron, rbcL and matK for the Citrus species reported on Table 2. Nucleotide alignments were performed with MEGA7 [41].

Molecular investigations were carried out by amplifying two chloroplast markers (the trnL gene intron and the psbA-trnH intergenic spacer) and the two nuclear internal transcribed spacers (ITS1 and ITS2). The primers pairs adopted to amplify either chloroplast or nuclear regions, along with the relative nucleotide sequences and the corresponding references, are supplied in Table 6.

For each chloroplast and nuclear marker, PCR amplifications were conducted in a volume of 20 μL, containing 15 ng of genomic DNA as a template, 1X AccuPrime Pfx Reaction Mix (Invitrogen, Thermo Fisher Scientific), primers to a final concentration of 0.2 μM each and 0.25 U of AccuPrime Pfx DNA Polymerase (Invitrogen, Thermo Fisher Scientific).

All PCR amplifications were performed in the GeneAmp 9700 PCR System (Applied Biosystems, Waltham, MA, USA). The experimental conditions for PCR amplification were as follows: 2 min at 95 ℃, followed by 40 cycles of 15 s at 95 ℃, 30 s at 55 ℃, 1 min at 68 ℃.

Positive and negative controls were used as reference standards. The PCR-derived fragments were resolved in 2% agarose/TAE gels and visualized under UV light via Sybr Safe DNA staining (Life Technologies, Carlsband, CA, USA).

Amplification products originated with chloroplast primer combinations (trnL-intron and psbA-trnH IGS) were subjected to EXOI/FAP (Thermo Scientific, Bartlesville, OK, USA) treatment and then directly sequenced on an ABI3100 automated sequencer. For the ITS 5.8S rRNA region, amplification products were purified by using the QIAquick PCR Purification Kit. Purified PCR products were adenylated in reaction volume of 10 μL containing 1X PCR buffer (100 mM Tris-HCl pH 9.0, 15 mM MgCl${}_{2}$ and 500 mM KCl), 0.2 mM dNTPs, and 0.5 U of Taq DNA polymerase (BIOLINE, London, UK). Adenylated amplicons were sub-cloned by using the kit StrataClone PCR Cloning Kit (Agilent, Santa Clara, CA, USA). and transformed into chemically competent StrataCloneSoloPack Competent Cells (Agilent). Bacteria were plated on LB plates (1.5% agar, 50 μg/mL ampicillin, 40 μg/mL X-Gal), and transformed colonies were selected by Colony-PCR. Amplification reactions were performed in a total volume of 20 μL including 2 μL of 10X reaction buffer, 1.5 mM MgCl${}_{2}$, 300 μM dNTPs, 1.5 U of BIOTaq DNA polymerase (BIOLINE), 0.2 μM of T3 and T7 primers. Positive colonies were grown over night on LB media. For each PCR product, 5 positive clones were selected by colony PCR. Plasmid were purified from positive clones by using the GenElute Plasmid Miniprep Kit (Sigma-Aldrich), by following the manufacturer instructions. The sequencing of PCR products (trnL-intron and psbA-trnH IGS) and plasmids (ITS 5.8S rRNA regions) was done by using an ABI3100 automated sequencer (Applied Biosystems). Sequences were visualized and manually edited with Geneious 5.4 to minimize any possible error during sequencing. Sequence similarity searches were performed using the BLASTn algorithm (http://www.ncbi.nlm.nih.gov/BLAST) against the NCBI nr nucleotide databases. cpDNA and nuDNA sequences were deposited in GenBank with accession numbers: KY656107-KY656138.

The character-based technique was employed to look for unique sets of diagnostic characters related to single species of Citrus. Rather than using hierarchies or distance trees, character-based analysis classifies taxonomic groups based on shared specific informative character states, SNPs or InDels, at either one or multiple nucleotide positions [46]. The BLASTn algorithm (http://www.ncbi.nlm.nih.gov/BLAST) was used to perform sequence similarity searches against the nr nucleotide databases of NCBI and assess the specificity of PCR amplifications. Separate data analyses were performed for each individual sequence and for the combined chloroplast datasets. Analysis of polymorphism distribution was performed using the DnaSP v.4 software [47] to generate a map containing haplotype data without considering sites with alignment gaps.

For a tree-based analysis, multiple sequence alignments were performed with the software MEGA 7 [41]. The same software was used to calculate interspecific genetic divergences according to the Kimura 2-parameter distance model [48]. Based on the pairwise nucleotide sequence divergences (Tamura Nei), the Maximum Likelihood (ML) tree was estimated starting from the haplotype sequences of each plant accession. A bootstrap analysis was conducted to measure the stability of the computed branches with 1000 resampling replicates.

## Figures and Tables

**Table 1:** Chromosome arm length measures as inferred from microphotographs using the Karyotype software [33]. Chromosome 4 is classified as sub-metacentric according to Levan [32] while other chromosomes as metacentric. S and L indicate Short and Long arms of chromosomes, respectively.

Chromosome ID	L	S	S/L	S/(L+S)
**1**	13.48	9.71	1.39	41.85
**2**	11.54	10.07	1.15	46.60
**3**	11.15	9.07	1.23	44.87
**4**	12.96	6.38	2.03	32.97
**5**	10.90	6.48	1.62	38.16
**6**	10.46	6.48	1.61	38.26
**7**	9.44	6.43	1.47	40.51
**8**	8.38	5.67	1.48	40.33
**9**	7.64	5.77	1.32	43.02

**Table 2:** List of accessions used in this study. The classification names are according to Swingle and Reece [36]. CRB is the Citrus INRA-CIRAD San Giuliano Corsica, France.

Species	Variety	Origin	Accession Code
*C. micrantha*	Micrantha	CRB Citrus1	SRA1115
*C. maxima*	Sans pepin pummelo	CRB Citrus	SRA710
*C. reticulata*	Cleopatra mandarin	CRB Citrus	ICVN0110273
*C. medica*	Diamante citron	CRB Citrus	SRA540
*C. medica*	Poncire commun citron	CRB Citrus	SRA701
*C. sinensis*	Olinda Valencia sweet orange	CRB Citrus	SRA18
*C. x aurantium*	Maroc sour orange	CRB Citrus	ICVN0110033
*C. x bergamia*	Castagnaro bergamot	CRB Citrus	SRA 612
*C. limon*	Femminello	CRB Citrus	SRA180
*Citrus* sp.	Rhobs el arsa citron	CRB Citrus	ICVN0110244
*Citrus* sp.	Pompia	Milis	M1
*Citrus* sp.	Pompia	Milis	M3
*Citrus* sp.	Pompia	Bitti B	B
*Citrus* sp.	Pompia	Oliena	O150
*Citrus* sp.	Pompia	Oliena	O8
*Citrus* sp.	Pompia	Siniscola	ME1
*Citrus* sp.	Pompia	Siniscola	S3
*Citrus* sp.	Pompia	Siniscola	T1
*Citrus* sp.	Pompia	Siniscola	T4
*Citrus* sp.	Pompia	Siniscola	T5

**Table 3:** Average genetic distance between taxa (below diagonal) and average net distance between taxa (above diagonal) based on ITS sequences. The distances were calculated taking into account the proportion of nucleotide (p) at which each pair of sequences being compared are different.

	*C. medica*	*C. maxima*	*C. reticulata*	*C. micrantha*	*C. sinensis*	*C. aurantium*	*C. limon*	*C. bergamia*	Rhobs el arsa	Pompia
*C. medica*	-	0.015	0.024	0.017	0.013	0.015	0.015	0.000	0.000	0.001
*C. maxima*	0.015	-	0.017	0.013	0.000	0.000	0.004	0.002	0.000	0.007
*C. reticulata*	0.024	0.017	-	0.022	0.013	0.017	0.013	0.011	0.013	0.019
*C.micrantha*	0.017	0.013	0.022	-	0.011	0.013	0.013	0.007	0.007	0.012
*C. sinensis*	0.016	0.003	0.016	0.014	-	0.000	0.004	0.001	−0.001	0.006
*C. aurantium*	0.015	0.000	0.017	0.013	0.003	-	0.004	0.002	0.000	0.007
*C. limon*	0.015	0.004	0.013	0.013	0.007	0.004	-	0.000	0.002	0.008
*C. bergamia*	0.007	0.009	0.019	0.015	0.011	0.009	0.007	-	−0.003	−0.003
Rhobs el arsa	0.007	0.007	0.021	0.015	0.009	0.007	0.009	0.008	-	−0.003
Pompia	0.004	0.010	0.022	0.016	0.012	0.010	0.012	0.008	0.007	-

**Table 4:** Average genetic distance between taxa at the psbA-trnH marker (below diagonal) and trnL-intron marker (above diagonal). The distances were calculated taking into account the proportion of nucleotide at which each pair of sequences being compared are different.

	*C. medica*	*C. maxima*	*C. reticulata*	*C. micrantha*	*C. sinensis*	*C. aurantium*	*C. limon*	*C. bergamia*	Rhobs el arsa	Pompia
*C. medica*	-	0.004	0.006	0.002	0.004	0.004	0.004	0.004	0.004	0.004
*C. maxima*	0.018	-	0.006	0.002	0.000	0.000	0.000	0.000	0.006	0.006
*C. reticulata*	0.009	0.009	-	0.004	0.006	0.006	0.006	0.006	0.006	0.006
*C. micrantha*	0.021	0.018	0.012	-	0.002	0.002	0.002	0.002	0.009	0.009
*C. sinensis*	0.016	0.002	0.007	0.016	-	0.000	0.000	0.000	0.000	0.000
*C. aurantium*	0.018	0.005	0.009	0.014	0.002	-	0.000	0.000	0.000	0.000
*C. limon*	0.018	0.005	0.009	0.014	0.002	0.000	-	0.000	0.000	0.000
*C. bergamia*	0.018	0.005	0.009	0.014	0.002	0.000	0.000	-	0.000	0.000
Rhobs el arsa	0.018	0.005	0.009	0.014	0.002	0.000	0.000	0.000	-	0.000
Pompia	0.018	0.005	0.009	0.014	0.002	0.000	0.000	0.000	0.000	-

**Table 5:** Haplotype identified at the ITS 1-2 locus in the analyzed Citrus spp. Hap1 was identified in Rhobs el arsa, Pompia and *C. aurantium*; Hap2 and Hap3 in *C. sinensis*; Hap4 in *C. maxima*; Hap5 in *C. bergamia*; Hap6 in *C. reticulata*; Hap7 in *C. micrantha*. Hap8 in Pompia, *C. medica*, Rhobs el arsa, *C. bergamia*; Hap9 in *C. limon*. The first row represents the alignment positions. The second reports the nucleotide at the corresponding alignment position. Monomorphic alignment positions are not shown ID means Insertion/Deletion. Alignment positions within the ITS1 are not underlined while those within ITS2 are underlined.

	11	37	71	72	73	76	77	83	100	108	130	132	138	154	211	212	213	214	215	216	217	218	219	220	221
Haplotypes	C	C	G	A	C	G	T	G	C	C	C	C	T	G	C	C	C	G	G	A	G	A	C	G	G
Hap1	.	.	.	.	.	.	.	.	.	.	.	.	T	.	.	.	.	.	.	.	.	.	.	.	.
Hap2	.	.	.	.	.	.	C	.	.	.	.	.	T	.	.	.	.	.	.	.	.	.	.	.	.
Hap3	.	.	.	.	.	.	.	.	.	.	.	.	T	.	.	.	.	.	.	.	.	.	.	.	.
Hap4	.	.	.	.	.	.	.	.	.	.	.	.	T	.	.	.	.	.	.	.	.	.	.	.	.
Hap5	.	.	.	.	.	.	.	.	.	T	.	.	.	.	.	.	.	.	.	.	.	.	.	.	.
Hap6	.	.	T	G	.	A	.	A	.	T	.	.	.	.	.	.	.	.	.	.	.	.	.	.	.
Hap7	.	.	.	.	T	.	.	.	T	.	.	.	.	.	.	.	.	.	.	.	.	.	.	.	.
Hap8	T	T	.	.	T	.	.	.	.	.	T	T	.	A	ID	ID	ID	ID	ID	ID	ID	ID	ID	ID	ID
Hap9	T	T	.	**G**	T	.	.	.	.	.	T	T	.	A	ID	ID	ID	ID	ID	ID	ID	ID	ID	ID	ID
	222	223	224	225	226	227	228	229	230	231	232	233	234	235	236	237	238	239	255	451	467	474	524	539	551
Haplotypes	T	G	C	G	C	T	G	C	G	G	G	G	T	G	C	G	G	T	T	ID	C	C	A	C	C
Hap1	.	.	.	.	.	.	.	.	.	.	.	.	.	.	.	.	.	T	.	ID	T	.	.	.	.
Hap2	.	.	.	.	.	.	.	.	.	.	.	.	.	.	.	.	.	.	.	ID	T	.	.	.	.
Hap3	.	.	.	.	.	.	.	.	.	.	.	.	.	.	.	.	.	.	.	ID	.	.	C	.	.
Hap4	.	.	.	.	.	.	.	.	.	.	.	.	.	.	.	.	.	.	.	ID	T	.	.	.	.
Hap5	.	.	.	.	.	C	.	.	.	.	.	.	.	.	.	.	.	.	.	ID	T	.	.	.	.
Hap6	.	.	.	.	.	C	.	.	.	.	.	.	.	.	.	.	.	.	.	ID	.	.	C	G	.
Hap7	.	.	.	.	.	C	.	.	.	.	.	.	.	.	.	.	.	.	C	A	.	T	.	.	T
Hap8	ID	ID	ID	ID	ID	ID	ID	ID	ID	ID	ID	ID	ID	ID	ID	ID	ID	ID	T	ID	.	.	.	.	.
Hap9	ID	ID	ID	ID	ID	ID	ID	ID	ID	ID	ID	ID	ID	ID	ID	ID	ID	ID	T	ID	.	.	.	.	.

**Table 6:** Primer list. For each nuclear and chloroplast marker, the amplicon length, primer names, primer sequences, annealing temperature and reference source are reported. P.w. means present work.

Marker	Amplicon Length	Primers	Primer Sequence		Ref.
ITS-5.8S rRNA	533–564, 534–563	ITS1	TCCGTWRGTGAACCWGCGG	54	[42]
		ITS4	TCCTCYRMTTAKYGATATGC	54	[42]
psbA-trnH IGS	442–468	psbA3f	GTTATGCATGAACGTAATGCTC	54	[43]
		trnHf	CGCATGGTGGATTCACAATCC	54	[44]
trnL intron	530–542, 536	trnLF	GGATAGGTGCAGAGACTCRATGGAAG	56	[45]
		trnLR	TGACATGTAGAATGGGACTCTATCTTTAT	56	[45]
ITS-5,8 SrRNA	241	Hap1-5 F	TGAAAGAAGGCACCGCACCC	66	P. w.
		Hap1-5 R	TCGAAACCTGCCCAGCAGAAC	66	P. w.
	194	Hap 8-9 F	GAAAGAAGGCGCCGCGGGA	68	P. w.
		Hap 8-9 R	GAACGACCCGTGAACCAGTTGATA	68	P. w.

## Supplementary Materials

The following are available online at https://www.mdpi.com/2223-7747/8/4/83/s1.

## Appendix A. Haplotypes at Plastidial Markers

### Appendix A.1. psbA-trnH Intergenic Spacer

**Table A1:** Haplotypes at the psbA-trnH intergenic spacer. The first row represent SNV position in the alignment while the second is the consensus sequence.

Position	44	93	103	111	113	137	155	172	177	178	179	180	181	182	183	184	186	219	227	230	231	232	233	234	235	236
Consensus	A	G	G	G	C	T	A	A	A	T	G	C	G	A	C	T	T	G	G	A	T	T	T	A	T	T
Hap1	.	.	.	A	.	.	.	.	ID	ID	ID	ID	ID	ID	ID	ID	.	T	G	.	.	.	.	.	.	.
Hap2	C	A	A	G	.	.	C	.	A	T	G	C	G	A	C	T	G	.	T	ID	ID	ID	ID	ID	ID	ID
Hap3	.	.	.	A	.	G	.	.	ID	ID	ID	ID	ID	ID	ID	ID	.	.	G	.	.	.	.	.	.	.
Hap4	.	.	.	G	.	.	.	.	A	T	G	C	G	A	C	T	.	.	T	.	.	.	.	.	.	.
Hap5	.	.	.	G	A	.	.	C	A	T	G	C	G	A	C	T	.	T	C	.	.	.	.	.	.	.
Hap6	.	.	.	A	.	.	.	.	ID	ID	ID	ID	ID	ID	ID	ID	.	.	G	.	.	.	.	.	.	.
Position	237	258	286	287	288	289	290	291	292	293	294	295	296	297	298	314	315	316	317	328	329	330	331	332	371	
Consensus	A	C	T	C	A	A	A	G	A	A	A	G	A	A	A	A	A	A	C	A	A	A	A	A	C	
Hap1	.	.	ID	ID	ID	ID	ID	ID	ID	ID	ID	ID	ID	ID	ID	A	A	A	C	.	.	.	.	.	.	
Hap2	ID	.	ID	ID	ID	ID	ID	ID	ID	ID	ID	ID	ID	ID	ID	ID	ID	ID	ID	ID	ID	ID	ID	ID	ID	
Hap3	.	.	ID	ID	ID	ID	ID	ID	ID	ID	ID	ID	ID	ID	ID	A	A	A	C	.	.	.	.	.	.	
Hap4	.	.	ID	ID	ID	ID	ID	ID	ID	ID	ID	ID	ID	ID	ID	ID	ID	ID	ID		.	.	.	.	.	
Hap5	.	.	T	C	A	A	A	G	A	A	A	G	A	A	A	ID	ID	ID	ID	.	.	.	.	ID	.	
Hap6	.	T	ID	ID	ID	ID	ID	ID	ID	ID	ID	ID	ID	ID	ID	A	A	A	C	.	.	.	.	ID	.	

### Appendix A.2. tnrL Intron

**Table A2:** Haplotypes at the tnrL intron. The first row represent SNV position in the alignment while the second is the consensus sequence.

Position	163	223	230	277	297	298	299	300	301	302	313	314	315	316	317	318
Consensus	A	G	T	T	A	G	A	A	A	A	T	G	T	T	A	T
Hap1	C	.	.	.	ID	ID	ID	ID	ID	ID						
Hap2	.	A	.	.	ID	ID	ID	ID	ID	ID	ID	ID	ID	ID	ID	ID
Hap3	C	.	.	.	ID	ID	ID	ID	ID	ID		.	.	.	.	
Hap4	.	.	G	C	.	.	.	.	.	.	.	.	.	.	.	.
Hap5	.	.	.	.	ID	ID	ID	ID	ID	ID		.	.	.	.	.
Hap6	C	.	.	.	ID	ID	ID	ID	ID	ID	.	.	.	.	.	.
