# Supplementary material for: Karyological Analysis and DNA Barcoding of Pompia Citron: A First Step toward the Identification of Its Relatives
Source: Plants (Basel). 2019 Mar 31;8(4):83. doi: 10.3390/plants8040083 (PMC6524030; doi:10.3390/plants8040083)
Supplement: Supplementary file 1 [file plants-08-00083-s001.zip › Supplemental/Supplemental_File1.docx]

### Supplemental material

**Supplementary File 1: Output Karyotype software**

Karyotype asymmetry degree (Stebbins, 1971): 2A

Karyotype formula (Levan et al., 1964): 2n = 2x = 16m + 2sm

Original Table (L: long arms; S: short arms; 0: no satellite; 1: on the long arm; 2: on the short arm; 3: intercalary satellite)

| **ID** | **L** | **S** | **L+S** | **L-S** | **L/S** | **Group ID** |
| --- | --- | --- | --- | --- | --- | --- |
| 1 | 11.34 | 9.16 | 20.50 | 2.18 | 1.24 | 3 |
| 2 | 11.54 | 10.02 | 21.56 | 1.52 | 1.15 | 2 |
| 3 | 14.04 | 10.24 | 24.28 | 3.80 | 1.37 | 1 |
| 4 | 12.93 | 9.16 | 22.09 | 3.77 | 1.41 | 1 |
| 5 | 12.61 | 6.68 | 19.29 | 5.92 | 1.89 | 4 |
| 6 | 10.90 | 6.53 | 17.43 | 4.37 | 1.67 | 5 |
| 7 | 10.26 | 6.43 | 16.69 | 3.83 | 1.60 | 6 |
| 8 | 8.34 | 5.90 | 14.25 | 2.44 | 1.41 | 8 |
| 9 | 7.48 | 6.04 | 13.52 | 1.44 | 1.24 | 9 |
| 10 | 11.54 | 10.12 | 21.66 | 1.42 | 1.14 | 2 |
| 11 | 10.90 | 6.92 | 17.82 | 3.98 | 1.57 | 5 |
| 12 | 9.79 | 6.53 | 16.32 | 3.26 | 1.50 | 7 |
| 13 | 9.10 | 6.34 | 15.43 | 2.76 | 1.44 | 7 |
| 14 | 10.96 | 8.98 | 19.94 | 1.97 | 1.22 | 3 |
| 15 | 10.65 | 6.53 | 17.18 | 4.12 | 1.63 | 6 |
| 16 | 7.81 | 5.51 | 13.32 | 2.31 | 1.42 | 9 |
| 17 | 13.32 | 6.07 | 19.39 | 7.25 | 2.19 | 4 |
| 18 | 8.42 | 5.43 | 13.85 | 2.99 | 1.55 | 8 |

| Other Information |  |  |  |  |  |  |
| --- | --- | --- | --- | --- | --- | --- |
| longest/shortest = 1.82 | |  |  |  |  |  |
| The total haploid length of the chromosome set (Peruzzi et al., 2009), THL = 162.25 | | | | | | |
| Coefficient of Variation of Centromeric Index (Paszko, 2006), CVCI = 9.94 | | | | | |  |
| Coefficient of Variation of Chromosome Length (Paszko, 2006), CVCL = 18.51 | | | | | | |
| Mean Centromeric Asymmetry (Peruzzi and Eroglu, 2013), MCA = 18.54 | | | | | |  |
| ------------------------------------------------------------- | | | |  |  |  |
| Number of chromosome which (long arm/short arm) > 2: 1 (5.56%) | | | | | |  |
| The Karyotype asymmetry index (Arano, 1963), AsK% = 59.14% | | | | | |  |
| The total form percent (Huziwara, 1962), TF% = 40.86% | | | | |  |  |
| The index of Karyotype symmetry (Greilhuber and Speta, 1976), Syi = 69.09% | | | | | | |
| The index of chromosomal size resemblance (Greilhuber and Speta, 1976), Rec = 74.26% | | | | | | |
| The intra chromosomalasymmetryindex (Romero Zarco, 1986), A1 = 0.31 | | | | | |  |
| The inter chromosomal asymmetry index( Romero Zarco, 1986), A2 = 0.19 | | | | | |  |
| The degree of asymmetry of Karyotype (Watanabe et al., 1999), A = 0.19 | | | | | |  |
| The dispersionindex (Lavania and Srivastava, 1992), DI = 6.80 | | | | |  |  |
| The asymmetryindex (Paszko, 2006), AI = 1.84 | | | |  |  |  |

**References:**

Arano H (1963) Cytological studies in subfamily Carduoideae (Compositae) of Japan. IX. The karyotype analysis and phylogenic considerations on Pertya and Ainsliaea. Botanical Magazine (Tokyo) 76: 32–39.

Greilhuber J, Speta F (1976) C-banded karyotypes in the Scilla hohenackeri Group, S. persica and Puschkinia (Liliaceae). Plant Systematics and Evolution 126: 149–188. doi: 10.1007/ BF00981669

Huziwara Y (1962) Karyotype analysis in some genera of Compositae. VIII. Further studies on the chromosome of Aster. American Journal of Botany 49: 116–119. doi: 10.2307/2439026

Lavania UC, Srivastava S (1992) A simple parameter of dispersion index that serves as a adjunct to karyotype asymmetry. Journal of Biosciences 17: 179–182. doi: 10.1007/BF02703503

Levan A, Fredga K, Sandberg AA (1964) Nomenclature for centromeric position on chromosomes. Hereditas 52: 201–220. doi: 10.1111/j.1601-5223.1964.tb01953.x

Paszko A (2006) A critical review and a new proposal of karyotype asymmetry indices. Plant Systematics and Evolution 258: 39–48. doi: 10.1007/s00606-005-0389-2

Peruzzi L, Leitch IJ, Caparelli KF (2009) Chromosome diversity and evolution in Liliaceae. Annals of Botany (London) 103: 459–475. doi: 10.1093/aob/mcn230

Peruzzi L., Eroglu HE (2013) Comparative analyss:again, how to measure ? Comp Cytogen 7(1),1-9

Romero Zarco C (1986) A new method for estimating karyotype asymmetry. Taxon 35: 526–530. doi: 10.2307/1221906

Stebbins GL (1971) Chromosomal evolution in higher plants. London, UK: Edward Arnold (Publishers) Ltd.

Watanabe K, Yahara T, Denda T, Kosuge K (1999) Chromosomal evolution in the genus Brachyscome (Asteraceae, Astereae): Statistical tests regarding correlation between changes in karyotype and habit using phylogenetic information. Journal of Plant Research 112: 145–161. doi: 10.1007/PL00013869
